# Systematic review of extracellular vesicle-derived microRNAs involved in organ fibrosis: implications for arthrofibrosis therapy

**DOI:** 10.1186/s12967-025-06810-x

**Published:** 2025-07-17

**Authors:** Venkateswaran Ganesh, Rui He, James A. Martin, Aliasger K. Salem, Edward A. Sander, Kyungsup Shin, Dongrim Seol

**Affiliations:** 1 Department of Orthopedics and Rehabilitation, Carver College of Medicine, University of Iowa, Iowa City, IA 52242 USA; 2 Roy J. Carver Department of Biomedical Engineering, College of Engineering, University of Iowa, Iowa City, IA 52242 USA; 3 Pharmaceutical Sciences and Experimental Therapeutics, College of Pharmacy, University of Iowa, Iowa City, IA 52242 USA; 4 Department of Orthodontics, College of Dentistry and Dental Clinics, University of Iowa, Iowa City, IA 52242 USA

**Keywords:** Arthrofibrosis, Fibrosis, Extracellular vesicles, Exosomes, MicroRNAs, Myofibroblasts

## Abstract

Arthrofibrosis is defined as the excessive accumulation of connective tissue in and around joints, which interferes with the range of motion required for activities of daily living. Although joint stiffness can be restored by surgical interventions such as adhesion lysis, arthroscopic debridement, and capsular release, arthrofibrosis tends to redevelop in the months following the surgery. Thus, there is a critical and urgent need to develop a non-invasive, pharmacological therapy to prevent or resolve arthrofibrosis. A subclass of small extracellular vesicles called exosomes convey bioactive regulators like micro ribonucleic acids (miRNAs/miRs), which can function as anti- and pro-fibrotic agents. Currently, there is no research on miRNA-based therapeutic potentials for treating arthrofibrosis. Previous research and clinical observations on fibrosis across organ systems suggest that there are commonalities in pathogenic mechanisms that can be targeted in arthrofibrosis therapy. In this study, we collated and critically analyzed the existing literature on exosomal miRNAs in organ fibrosis to discover potential candidates for diagnosing, preventing, and/or treating arthrofibrosis. Fifty-six articles were finally selected and categorized by anti- and pro-fibrotic candidates of miRNAs. Notably, let-7, miR-26, miR-29, miR-146, miR-148/-152, miR-214, miR-223, and miR-21 emerged as prominent candidates that should be investigated further for effectiveness in arthrofibrosis therapy.

## Introduction

Arthrofibrosis (AF) is the excessive deposition of fibrous tissue by intra- and peri-articular tissue cells, leading to joint pain and restricted range of motion (ROM). Annually, an alarming average of three million patients in the United States report abnormal stiffness after a joint insult from traumatic injury, surgery, or prolonged immobilization [1]. Conservative management practices like guided physiotherapy, corticosteroids, and splinting/bracing are often prescribed to limit relatively mild AF symptoms [2, 3]. Surgery to remove adhesions or to release capsular contractures is employed in more severe cases, but these interventions often do not fully restore normal ROM, and fibrosis tends to redevelop in the months following surgery [4, 5]. Consequently, there is an urgent need to develop pharmaceutical approaches to augment surgical and managerial approaches that improve ROM gains and overall patient quality of life.

### Current pharmaceutical treatments in fibrosis

The characteristic thickening of the joint capsule associated with AF is thought to occur as part of the same aberrant wound-healing response that underlies the basic mechanisms of soft tissue fibrosis. This idea is further motivated by reports on the abundance of myofibroblasts in the contracted tissue. The characteristic of collagen-rich extracellular matrix (ECM) under high mechanical tension common in fibrotic soft tissues is often attributed in part to the presence of myofibroblast-like cell phenotypes that exhibit high levels of contractile activity. Myofibroblasts derive from multiple precursors, including but not limited to fibroblasts, pericytes, fibrocytes, epithelial, and endothelial cells [6]. While they are often transiently involved in normal wound healing, fibrosis is characterized by their abnormal persistence post-injury. As a result, many promising strategies to restore normal ECM architecture often target the differentiation and survival of these cells [7, 8]. Myofibroblast persistence in fibrotic tissues is driven in part by chronic inflammation, which promotes and maintains the myofibroblast phenotype [9, 10]. This explains why corticosteroids and nonsteroidal anti-inflammatory drugs (NSAIDs) are often prescribed as anti-fibrotic drugs. Likewise, drugs that directly target myofibroblast contractility by relaxing cytoskeletal tension, and drugs that minimize myofibroblast differentiation and survival show great promise as anti-fibrotic therapies [11–13]. 

### Exosomal micro ribonucleic acids (miRNAs/miRs)

Exosomes are nanosized lipid membrane-bound extracellular vesicles (30–150 nm) secreted by all living cells. They are now recognized to play a central role in intercellular communication [14]. Exosomes carry complex cargoes of bioactive molecules such as proteins, lipids, and nucleic acids including miRNAs, a class of short (~ 22 nucleotides) non-coding regulatory RNAs that modulate over 60% of protein-encoding gene expression when internalized by recipient cells [15, 16]. These miRNAs regulate a multitude of cellular pathways related to inflammation [17, 18], differentiation [19], ECM composition [20], and apoptosis [21]. miRNAs act by binding to specific messenger RNAs (mRNAs) *via* sequence homology. The formation of such miRNA/mRNA complexes can accelerate mRNA decay or repress translation [22–24]. 

The lipid membrane surrounding exosomes protects miRNAs from degradation by extracellular nucleases and greatly enhances the efficiency of transfer from donor to recipient cells [25]. miRNAs originate inside the nucleus as transcribed precursor miRNAs (pre-miRNAs) and reach maturity outside in the cytosol [26, 27]. miRNAs are commonly cataloged into respective families based on sequence similarity. Genes encoding miRNA family members are often organized in clusters and are co-transcribed [28, 29]. 

Precursor miRNAs are initially transcribed in the nucleus and undergo site-specific cleavage to produce mature miRNAs in the cytoplasm. The latter process can generate subtle differences in nucleotide sequences among family members that affect mRNA binding. This can result in multiple miRNA sequences acting in concert to target the same mRNA. In contrast, a single miRNA can target multiple mRNAs [30], as well as circular RNAs (circRNAs) [31], and transfer RNAs (tRNAs) [32]. Difficulties in understanding how miRNAs regulate their various targets are further compounded when one also considers that they are delivered *via* exosomes in both an autocrine and paracrine fashion along with multiple other bioactive species. This review focuses on miRNAs reported for substantial activity in organ fibrosis, with the goal of identifying candidate miRNAs for AF treatment.

Despite mounting evidence that some miRNAs have anti-fibrotic properties in other organs, their therapeutic potential for the treatment of AF remains largely unexplored. Moreover, hundreds of peer-reviewed primary journals over the past few years have reported the effectiveness of miRNAs in soft tissue scarring of various organs. However, no comprehensive summaries of this unique niche currently exist. To address this issue, we systematically reviewed studies involving exosomal miRNA to identify sequences that target inflammation and excessive ECM deposition, the two classical indicators of fibrogenesis. Tabulating preclinical outcomes of exosomal miRNA treatment in scarring should delineate the molecular pattern of action, and this compendium is intended to serve as a guide for selecting a repertoire of miRNAs encapsulated in exosomes for novel anti-AF therapies.

## Methods

**Fig. 1:**
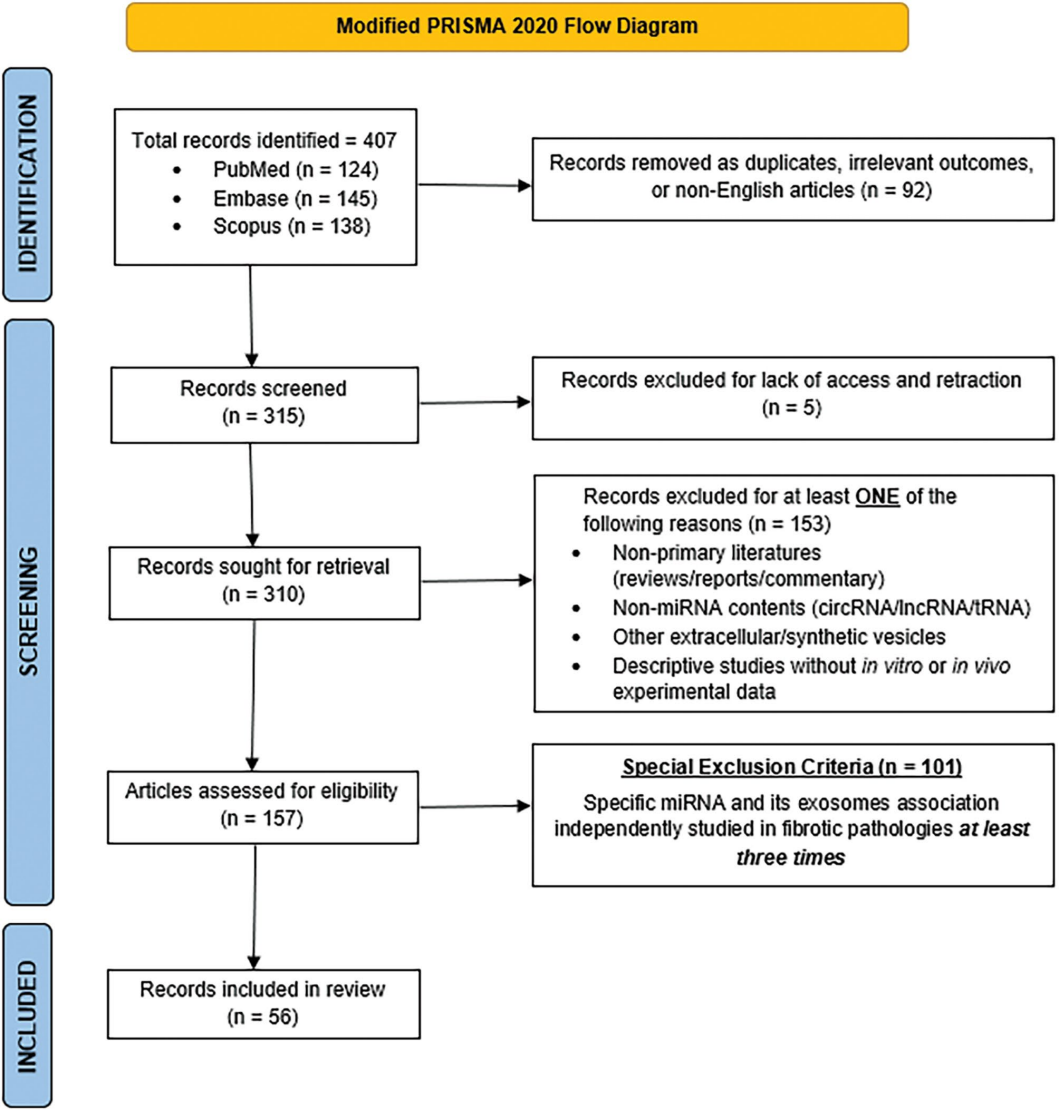
Modified PRISMA flow diagram for systemic reviews [33]. miRNA: micro ribonucleic acids, IncRNA: long non-coding RNA, tRNA: transfer RNA. Special Exclusion Criteria: exosomal miRNAs with less than 3 publications supporting association with fibrotic pathologies were excluded

A search was devised in October 2023 to locate articles relevant to this study according to the Preferred Reporting Items for Systematic Review and Meta-Analysis (PRISMA) guideline (Fig. 1) [33]. To identify records with concepts of common interest in Embase, PubMed, and Scopus databases, the “AND” function was used to combine the following subject terms: fibrosis, exosomes, and miRNAs. Two authors (V.G. and D.S.) independently performed data extraction and a third author (J.A.M.) involved discrepancy resolution. Following the removal of duplicates, all three databases together yielded 315 records. These records were further refined by applying the following exclusion criteria: (i) non-miRNA exosomal cargo; (ii) theoretical studies without experimental validation; and (iii) editorial or review articles. miRNAs with fewer than 3 publications documenting involvement in fibrotic conditions were excluded from consideration (*n* = 101) in order to limit the search to a more tractable set consisting of the most promising candidates for AF therapy. Applying these criteria resulted in fifty-six articles describing miRNAs that were further categorized as having either anti-fibrotic or pro-fibrotic characteristics.

## Results

Based on our search criteria, 8 miRNAs were identified: 7 for their anti-fibrotic potential (Sect. 3.1; Table 1) and 1 for their profibrotic potential (Sect. 3.2; Table 2). miRNAs having both anti- and pro-fibrotic effects are listed in Sect. 3.3 (Table 3). Below we summarize each of these miRNAs in alpha-numeric order.

**Table 1:** Candidates of anti-fibrotic Exosomal MicroRNAs (miRs) for arthrofibrosis treatment.

miRNAs	Target	Source of exosomes	Model		Pro/Anti -fibrosis	Outcomes	Ref.
			In vitro	In vivo			
[let-7 family]							
let-7d	Lung	Mouse BALF from PF	TGF-β1-induced mouse lung pericytes	Mouse PF model *via* bleomycin	Anti-	Suppressed Col1A and α-SMA by inhibiting TGF-βR1/FoxM1/Smad3/β-catenin	[36]
let-7	Lung	Human MenSCs	TGF-β1-induced MLE-12 cells	Mouse PF model *via* bleomycin	Anti-	Reduced fibrosis score, collagen deposition, and GSH-Px by inhibiting LOX1/NLRP3/caspase 3	[37]
let-7	Liver	Human liver stem cells	TNF-α- or TGF-β1-induced cholangiocytes (H69)	MDR2 ^**KO**^ mouse	Anti-	Reduced ductular reaction and biliary fibrosis by inhibiting IL-13 and NF-κB	[94]
let-7c	Kidney	Human BMSCs transfected with let-7c	TGF-β1-induced NRK-52E cells	Mouse RF model *via* UUO	Anti-	Attenuated kidney injury with downregulated Col4α1, MMP9, and TGF-β1/TGF-βR1	[95]
let-7i-5p	Kidney	BMSCs trans-fected with let-7i-5p antagomir	TGF-β1-induced NRK-52E cells	Mouse RF model *via* UUO	Pro-	Reduced renal fibrosis and improved kidney function by activating TSC1/mTOR	[38]
[miR-26 family]							
miR-26a-5p	Lung	HUMSCs transfected with miR-26a-5p	TGF-β1-induced MLE-12 cells	Mouse PF model *via* silica	Anti-	Suppressed EMT by inhibiting ADAM17/Notch	[41]
miR-26a	Kidney	HEK293 transfected with miR-26a	mTECs	Mouse RF model *via* alderstone	Anti-	Alleviated lipocalin 2, α-SMA, Col1, and fibronectin by inhibiting CTGF/Smad3	[42]
miR-26a	Kidney	HEK293 trans-fected with miR-26a precursor	MSMSCs	Mouse RF model *via* UUO	Anti-	Decreased CTGF and TGF-β1 by inhibiting FoxO1	[45]
miR-26a-5p	Heart	MSMSCs transfected with miR-26a-5p	MSMSCs and H9C2	Mouse chronic kidney disease model	Anti-	Depressed cardiac fibrosis with low expression of FoxO1, CTGF, fibronectin, and Col1A1	[96]
[miR-29 family]							
miR-29b-3p	Lung	Human BMSCs	Pulmonary fibroblasts (LL29)	Mouse PF model *via* bleomycin	Anti-	Suppressed fibroblast proliferation by downregulating FZD6	[46]
miR-29a/b/c-3p	Kidney	Mouse satellite cells transduced with Ad-miR29	NA	Mouce RF model *via* UUO	Anti-	Reduced TGF-β, α-SMA, fibronectin, and Col1A1 by inhibiting YY1 and TGF-β3.	[49]
miR-29b-3p	Kidney	Human liver stem cells	mTECs and mkCFs	Mouse AAN model *via* AA	Anti-	Prevented fibroblast activation by interfering β-catenin	[47]
miR-29b-3p	Heart	Rat BMSCs	NA	Rat MF model *via* MI	Anti-	Reduced MF and collagen volume fraction by inhibiting ADAMTS16	[48]
miR-29b	Heart	HUMSCs loaded with miR-29b	TGF-β1-induced cardiac fibroblasts	Mouse MF model *via* MI	Anti-	Alleviated inflammation and fibrosis, and improved cardiac function	[51]
miR-29a	Uterus	Human BMSCs	Mouse endometrial epithelial cells	Mouse IUA model	Anti-	Endometrium repair with reduced α-SMA, Col1, and Smad2/3	[97]
miR-29a	Skin	Human ADSCs transfected with miR-29a	human hypertrophic scar fibroblasts	Mouse thermal model	Anti-	Reduced excessive scar formation by inhibiting TGF-β2/Smad3	[50]
[miR-146 family]							
miR-146a-5p	Liver	Human and mouse serum	NA	Mouse schisto-somiasis model	Anti-	Low level of miR-146-5p in LF mice and patients.	[52]
miR-146a	Heart	Rat ADSCs transfected with miR-146a	hypoxia-induced cardiomyoblasts (H9c2)	Rat MF model *via* MI	Anti-	Suppressed apoptosis, inflammatory response, and fibrosis by downregulation of EGR1	[53]
miR-146a-5p	Heart	Human CPCs	Rat neonatal cardiomyocytes	Rat MF model *via* Dox/Trz	Anti-	Prevented MF, inflammatory cell infiltrate, and ventricular dysfunction	[54]
miR-146a	Urethra	HUMSCs treated with TNF-α	TGF-β1-induced urethral fibroblasts	Rat urethral fibrosis model *via* TGF-β1	Anti-	Suppressed urethral fibrosis, stricture, fibroblast activation, and inflammation	[55]
[miR-148 / -152 family]							
miR-148a	Liver	HUMSCs	BMDM and RAW264.7	Mouse LF model *via* CCI4	Anti-	Alleviated LF with macrophages by inhibiting KLF6/STAT3	[59]
miR-148a-5p	Liver	Mice BMSCs transfected with miR-148a-5p	TGF-β1-induced HSCs	Mouse LF model *via* thioacetamide	Anti-	Reduced TGF-β1, TIMP-1, Col1, and α-SMA levels by inhibiting Smad4	[58]
miR‑152‑5p	Heart	Human venous plasma from AMI patients	H9C2 transfected with miR-152-5p and its inhibitor	NA	Anti-	Increased fibrosis, apoptotic proteins when used miR-152-5p inhibitor by activating ARHGAP6/ROCK	[60]
[miR-214 family]							
miR-214	Liver	Mouse HSCs transfected with miR-21	Mouse hepatocytes, human HSCs and hepatocytes	NA	Anti-	Loss of miR-214 expression promotes CTGF-driven fibrosis (ColA1, α-SMA)	[62]
miR-214	Uterus	Human ESCs transfected with miR-214	TGF-β1-induced ESCs and epithelial cells	Mouse endometriosis model	Anti-	Reduced CTGF and fibrotic proteins	[63, 64]
[miR-223 family]							
miR-223-3p	Lung	HUMSCs	Silica-induced RAW264.7 and NIH/3T3 cell lines	Mouse PF model *via* silica	Anti-	Attenuated inflammatory and fibrosis factors by suppressing NLRP3 and circPWWP2A	[66]
miR-223	Liver	IL-6/PA-treated myeloid cells	Mouse hepatocytes (AML12)	Mouse IL-6RA^KO^ with high-fat diet	Anti-	Reduced expression of anti-fibrotic miR-223 in the LF model	[68]
miR-223	Heart	HUMSCs transfected with miR-223	TGF-β1-induced human cardiac fibroblasts	Mouse MF model *via* MI	Anti-	Relieved MF and inflammation *via* P53/S100A9 axis	[67]

(Abbreviation) AA: aristolochic acid, AAN: aristolochic acid nephropathy, Ad: adenovirus, ADAMTS: a disintegrin and metalloproteinase (ADAM) with thrombospondin motifs, ADSCs: adipose-derived mesenchymal stem cells, AMI: acute myocardial infarction, ARHGAP: Rho GTPase-activating protein, BALF: bronchoalveolar lavage fluid, BMDM: bone morrow-derived macrophages, BMSCs: bone marrow-derived mesenchymal stem cells, CCI4: carbon tetrachloride, circ: circular RNA, Col: collagen, CPCs: cardiac-resident mesenchymal progenitor cells, CTGF: connective tissue growth factor, Dox: doxorubicin, EGR: early growth response, EMT: epithelial to mesenchymal transition, ESCs: endometrial stromal cells, Fox: forkhead box, FZD: frizzled, GSH-Px: glutathione peroxidase, H9C2: cardiac myoblasts, HEK: human embryonic kidney, HSCs: hepatic stellate cells, HUMSCs: human umbilical cord mesenchymal stem cells, IL: interleukin, IUA: intrauterine adhesion, KLF: Krüppel-like factor, KO: knockout, Let: lethal, LF: liver fibrosis, LOX: lectin-like oxidized low-density lipoprotein receptor, MDR: multidrug resistance protein, MenSCs: menstrual blood-derived stem cells, MF: myocardial fibrosis, MI: myocardial infarction, mkCFs: mouse kidney cortical fibroblasts, MLE: murine lung epithelial, MMP: matrix metalloproteinase, MSMSCs: mouse skeletal muscle satellite cells, mTECs: mouse tubular epithelial cells, mTOR: mammalian target of rapamycin, NA: not applicable, NF: nuclear factor, NIH/3T3: murine fibroblast cell line, NLRP: nucleotide-binding and oligomerization domain-like receptor family pyrin domain-containing, Notch: neurogenic locus notch homolog protein, NRK: neighboring rat kidney tubular epithelial, PA: palmitic acid, PF: pulmonary fibrosis, RA: receptor A, RAW264.7: mouse macrophages, RF: renal fibrosis, ROCK: Rho-associated coiled-coil containing kinase, SMA: smooth muscle actin, Smad: suppressor of mothers against decapentaplegic, STAT: signal transducer and activator of transcription, TGF: transforming growth factor, TIMP: tissue inhibitor of metalloproteinase, TNF: tumor necrosis factor, Trz: trastuzumab, TSC: tuberous sclerosis complex, UUO: unilateral ureteral obstruction, YY: transcription factor Yin Yang

**Table 2:** A candidate of pro-fibrotic Exosomal microRNA-21 (miR-21) for arthrofibrosis treatment.

miRNAs	Target	Source of exosomes	Model		Pro/Anti -fibrosis	Outcomes	Ref.
			In vitro	In vivo			
[miR-21 family]							
miR-21	Lung	Arsenic-treated pulmonary epithelial cells	MRC-5 cells	miR-21^**KO**^ mouse model	Pro-	Blocked PF by inhibiting Akt activation and glycolysis in miR-21 ^**KO**^ mouse	[70]
miR-21	Lung	HBE	HBE and MRC-5 cells	Mouse PF model *via* CS	Pro-	Up-regulated miR-21 by activating TGF-β1/Smad3 in CS-induced PF	[71]
miR-21-5p	Lung	Fibrocytes from fibrotic lungs	MRC-5 cells and fibrocytes	Rat RF model *via* TGF-β1	Pro-	Increased miR-21-5p expression in fibrotic cells and patients	[73]
miR-21-5p	Lung	Human and mouse serum	NA	Mouse PF model *via* bleomycin	Pro-	Elevated miR-21 expression in serum exosomes	[72]
miR-21-5p	Lung	Rat BALF	293 T cell line	Rat RF model *via* coal dust particles	Pro-	Over-expressed miR-21-5p in rat BALF by suppressing Smad7	[98]
miR-21	Kidney	HPTCs	HPTCs	Rat calorific restriction model	Pro-	Prevented the occurrence of EMT by inhibiting miR-21	[74]
miR-21	Kidney	TGF-β1-stimulated rat NRK-52E cells	Rat NRK-49 F cells	Mouse RF model *via* UUO and Rab27a^KO^ model	Pro-	Activated fibroblasts and aggravated RF by PTEN/Akt in RF model and vice versa in KO model	[75]
miR-21a-5p	Kidney	Mouse BMSCs	Tubular epithelial cells	Mouse RF model *via* UUO	Anti-	Alleviated RF by attenuating glycolysis and PFKM	[76]
miR-21	Heart	HPB transfected with miR-21	hypoxia-induced cardiac muscle cells	Mouse MF model *via* MI	Pro-	Enhanced MF when treated with miR-21 mimic-loaded exosomes	[99]
miR-21-3p	Heart	Rat cardiac fibroblasts	Rat cardiomyocytes	Mouse CH model *via* Angiotensin II	Pro-	Attenuated CH by miR-21 inhibitor	[100]
miR-21	Pancreas	Mouse PSCs transfected with miR-21	Rat SAM-K PSCs	Mouse chronic pancreatitis model *via* cerulein	Pro-	Enhanced expression of miR-21 and Col1A	[101]
miR-21a-3p	Tendon	HUMSCs	TGF-β1-induced rat fibroblast cells	Rat Achilles tendon injury model	Pro-	Inhibited tendon adhesion when treated with low miR-21-3p-expressed HUMSC exosomes	[78]

(Abbreviation) Akt: protein kinase B, BALF bronchoalveolar lavage fluid, BMSCs: bone marrow-derived mesenchymal stem cells, CH: cardiac hypertrophy, Col: collagen, CS: cigarette smoke, EMT: epithelial to mesenchymal transition, HBE: human bronchial epithelial, HPB: human peripheral blood, HPTCs: human proximal tubular cells, HUMSCs: human umbilical cord mesenchymal stem cells, KO: knockout, MF: myocardial fibrosis, MI: myocardial infarction, MRC-5: human embryonic lung fibroblast, NA: not applicable, NRK: neighboring rat kidney tubular epithelial, PF: pulmonary fibrosis, PFKM: phosphofructokinase muscle isoform, PSCs: pancreatic stellate cells, PTEN: phosphatase and tensin homolog, RF: renal fibrosis, Smad: suppressor of mothers against decapentaplegic, TGF: transforming growth factor, UUO: unilateral ureteral obstruction

**Table 3:** Other candidates of Exosomal MicroRNAs (miRs) for arthrofibrosis treatment.

miRNAs	Target	Source of exosomes	Model		Pro/Anti -fibrosis	Outcomes	Ref.
			In vitro	In vivo			
[miR-122 family]							
miR-122	Liver	ADSCs transfected with miR-122	Human HSCs	Mouse LF model *via* CCI4	Anti-	Suppressed the activation of HSCs and collagen deposition	[82]
miR-122	Liver	Rat hepatic and plasma from LF	NA	Rat LF model *via* CCI4	Anti-	Suppressed pro-fibrotic miRNAs by PDE5 inhibitor	[102]
miR-122	Liver	PA-treated Huh-7 and serum from NAFLD patients	Human HSCs	NA	Pro-	Enhanced expression of fibrosis markers including Col1A1, α-SMA, and TGF-β1	[88]
miR-122a	Kidney	Human BMSCs transfected with miR-122a	TGF-β1-induced HK-2	Rat RF model *via* UUO	Anti-	Reduced expansion of renal tubule and interstitial expansion by inhibiting mTOR and autophagy	[83]
[miR-192 family]							
miR-192	Liver	JFH-1 and Huh-7 cells transfected with miR-192	Human HSCs	NA	Pro-	Upregulated fibrogenic markers and activated trans-differentiation of HSCs	[103]
miR-192	Liver	PA-treated Huh-7 and serum from NAFLD patients	Human HSCs	NA	Pro-	Enhanced expression of fibrosis markers including Col1A1, α-SMA, and TGF-β1	[88]
miR-192-5p	Liver	Human ADSCs	Human hypertrophic scar fibroblasts	Mouse full-thickness skin defect model	Anti-	Attenuated hypertrophic scar formation and trans-differentiation by inhibiting IL-17RA/Smad	[79]
miR-192-5p	Liver	Human and rat serum from NAFLD	NA	Rat with high-fat/-cholesterol diet	Pro-	Activated proinflammatory macrophages and NAFLD by inhibiting Rictor/Akt/FoxO1 axis	[89]
[miR-150 family]							
miR-150-5p	Liver	Mouse ADSCs	TGF-β1-induced mouse HSCs	Mouse LF model *via* CCI4	Anti-	Attenuated hepatic fibrosis by inhibiting CXCL1	[80]
miR-150	Kidney	Hypoxia mTECs (NRK-52E)	Mouse NRK-49 F cells	Mouse RF model *via* IR	Pro-	Developed more pro-fibrotic manifestations by inhibiting SOCS1	[86, 87]
[miR-182 family]							
miR-181-5p	Liver	Mouse ADSCs transfected with miR-181-5p	TGF-β1-induced HST-T6 cells	Mouse LF model *via* CCI4	Anti-	Reduced Col1, vimentin, α-SMA, and fibronectin by inhibiting STAT3 and Bcl-2	[84]
miR-181a-2-3p	Liver	L-02 cells treated with citreoviridin	HSCs	ICR mouse model	Pro-	Reduced mitochondrial calcium accumulation and HSC activation by miR-181a-2-3p-antagomir	[90]
miR-181d	Kidney	Human BMSCs	TGF-β1-induced HK-2 cells	Rat RF model *via* UUO	Anti-	Restricted RF, Col4 α1/1, α-SMA, and TGF-βR1 by inhibiting KLF6 and NF-κB	[81]
miR-181a	Heart	Hypoxia-induced iCMs	Hypoxia-induced iCMs	Rat MF model *via* MI	Pro-	Attenuated MF and hypertrophy by miR-181a antagomir	[85]

(Abbreviation) ADSCs: adipose-derived mesenchymal stem cells, Akt: protein kinase B, Bcl-2: B-cell lymphoma 2, BMSCs: bone marrow-derived mesenchymal stem cells, CCI4: carbon tetrachloride, Col: collagen, CXCL: C-X-C motif chemokine ligand, Fox: forkhead box, HK: human renal tubular epithelial cells, HSCs: hepatic stellate cells, HST-T6: hepatic stellate, Huh-7: human hepatoma cells, iCMs: human induced pluripotent stem cell-derived cardiomyocytes, ICR: Institute of Cancer Research, IL: interleukin, IR: ischemia-reperfusion, JFH-1: hepatitis C virus genotype 2a, KLF: Krüppel-like factor, L-02: normal hepatocytes, LF: liver fibrosis, MF: myocardial fibrosis, MI: myocardial infarction, mTOR: mammalian target of rapamycin, NA: not applicable, NAFLD: non-alcoholic fatty liver disease, NF: nuclear factor, NRK: neighboring rat kidney tubular epithelial, PA: palmitic acid, PDE: phosphodiesterase, RA: receptor A, RF: renal fibrosis, Rictor: rapamycin-insensitive companion of mammalian target of rapamycin, SMA: smooth muscle actin, Smad: suppressor of mothers against decapentaplegic, SOCS: suppressor of cytokine signaling, STAT: signal transducer and activator of transcription, TGF: transforming growth factor, UUO: unilateral ureteral obstruction

### Anti-fibrotic candidates

#### Let-7

Let-7 is one of the largest miRNA families with anti-fibrotic potential, as all but one of its five members suppress TGF-β1-driven induction of myofibroblast differentiation [34, 35]. For example, it was found that exosomes from bronchoalveolar lavage fluid (BALF) with low let-7d enhanced lung pericyte differentiation into myofibroblasts, as evidenced by increases in collagen type 1 (COL1) and alpha smooth muscle actin (α-SMA) expression *via* a TGF-β receptor 1 (TGF-βR1)-dependent pathway [36]. Exosomal let-7 from menstrual blood-derived stem cells alleviated lung fibrosis in mice treated with bleomycin by suppressing reactive oxygen species (ROS) production and mitochondrial deoxyribonucleic acid (DNA) damage [37]. In multidrug resistance 2 gene knockout (MDR2 KO) mice, liver stem cell exosomes carrying let-7a/c improved liver scarring by decreasing Lin28a/b, nuclear factor kappa B (NF-κB), and nuclear receptor subfamily 1 group H member 4 (NR1H4) activation in cholangiocytes. In a unilateral ureteral obstruction (UUO) model, an antagomir targeting let-7i-5p reduced renal fibrosis by limiting collagen and fibronectin secretion of cells undergoing epithelial-mesenchymal transition (EMT) *via* inhibition of tuberous sclerosis complex 1 (TSC1) and mammalian target of rapamycin (mTOR) pathways [38]. This finding suggests that unlike other members of the let-7 family, let-7i-5p is pro-fibrotic.

#### miR-26

**Fig. 2:**
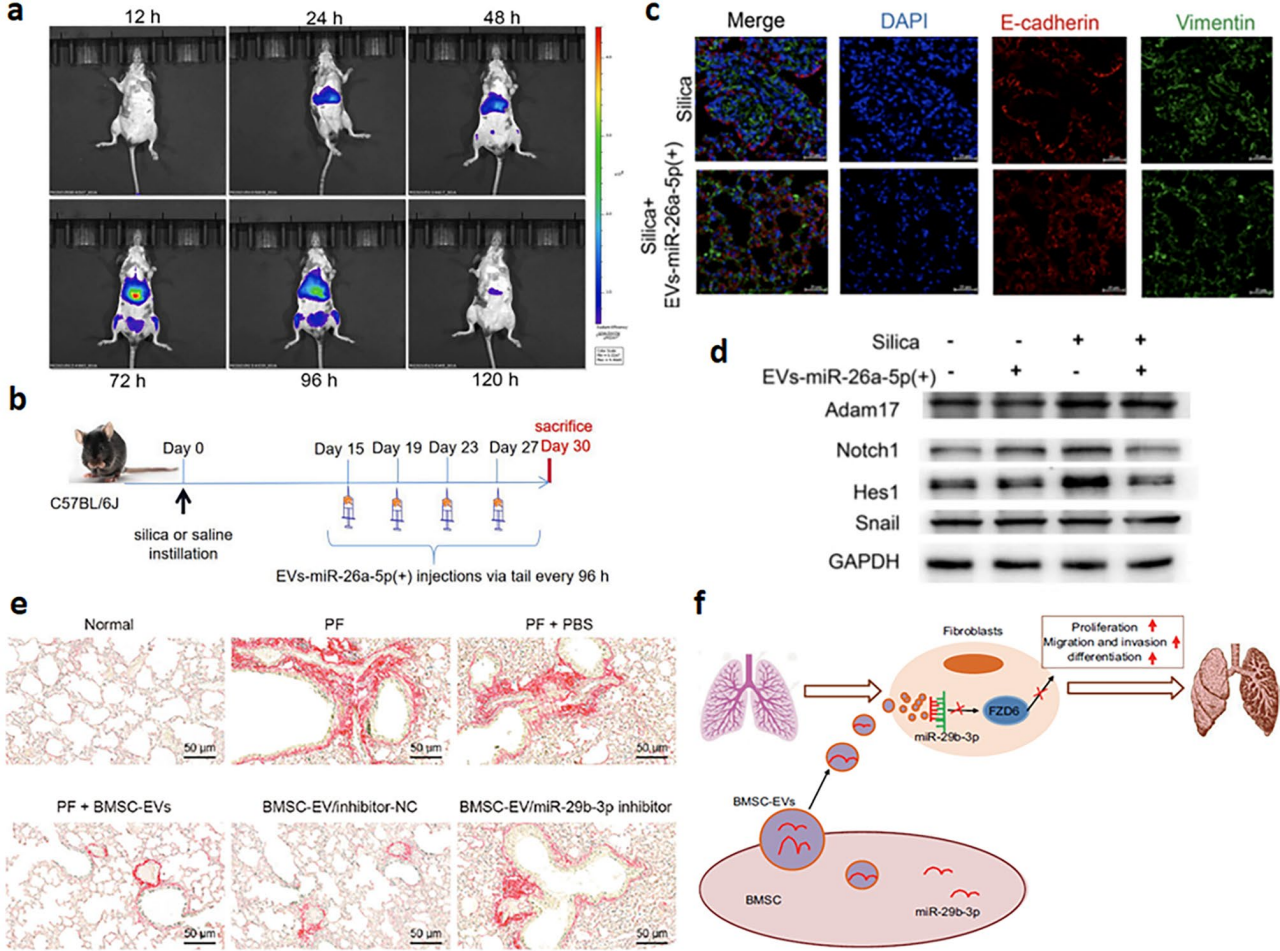
Anti-fibrotic effects of exosomal micro ribonucleic acids (miRs/miRNAs) in fibrous tissues. (**A-D**) EVs-miR-26a-5p(+) improved the respiratory function damaged by silica in mice. (**A**) Fluorescent signals of DiR-labeled EVs-miR-26a-5p(+). (**B**) A schematic diagram for experimental design. (**C**) Immunofluorescence staining in lung tissue. (**D**) Western blotting analysis of a disintegrin and metalloproteinase domain-containing protein 17 (Adam17), neurogenic locus notch homolog protein 1 (Notch1), hairy and enhancer of split 1 (Hes1), and glyceraldehyde-3-phosphate dehydrogenase (GAPDH) [41]. Copyright 2023, Elsevier Inc. (**E** and **F**) Bone marrow-derived mesenchymal stem cells-secreted extracellular vesicles (BMSC-EVs) containing miR-29b-3p inhibit the expression of Frizzled 6 (FZD6) and improve pulmonary fibrosis (PF). (**E**) Sirius Red staining of mouse pulmonary tissues. (**F**) Summary of interactions between BMSC-EVs containing miR-29b-3p and PF. PBS: phosphate-buffered saline, NC: negative control [46]. Copyright 2020, Wiley Periodicals LLC

miR-26 is another family of anti-fibrotic miRNAs that appear to oppose myofibroblast differentiation and inflammatory responses that precede or accompany scar establishment [39, 40]. In a silica/TGF-induced lung fibrosis model, exosomal miR-26a-5p from human umbilical cord mesenchymal stem cells (HUMSCs) disrupted a disintegrin and metalloproteinase domain-containing protein 17 (ADAM17)/neurogenic locus notch homolog protein (Notch) axis of EMT to lessen scarring in vivo (Fig. 2A-D) [41]. miR-26a also inhibited suppressor of mothers against decapentaplegic homolog 3 (Smad3) activation and reduced EMT in aldosterone-induced tubulointerstitial fibrosis [42]. Here, connective tissue growth factor (CTGF) expression, a prominent downstream effector of TGF-β1 signaling known to promote myofibroblast survival [43, 44], was suppressed by exosomal miR-26a to limit muscle atrophy *via* forkhead box protein O1 (FoxO1)/glycogen synthase kinase 3β (GSK-3β) in an obstructive kidney disease model [45]. Clearly, the miRNA-26 family has potential as an anti-fibrotic treatment.

#### miR-29

In organs like the heart, lung, kidney, uterus, and skin, miR-29 members are vital in modulating fibrotic gene expressions from pathologic TGF-β signaling. Bone marrow-derived mesenchymal stem cell (BMSC) exosomes loaded with miR-29b-3p inhibited fibroblast activation by targeting frizzled-6 (FDZ6), a receptor of the wingless-type mouse mammary tumor virus integration site family member 4 (Wnt4) ligand critical for cell differentiation in the mouse idiopathic pulmonary fibrosis (IPF) model (Fig. 2E and F) [46]. Liver stem cell-derived exosomes with miR-29a/b/c interfered with Wnt signaling and α-SMA expression in TGF-β1-treated fibroblasts [47]. These anti-fibrotic properties also were observed in a rat myocardial infarction model where miR-29b-3 targeted ADAM with thrombospondin motifs 16 (ADAMTS16), a promoter of cardiac myofibroblasts that regulates angiogenic ventricular remodeling [48]. 

Bioengineering exosomes permits one to not only specify the cargo but also to modify the surface recognition site for more efficient delivery to target cells. In renal fibrosis, surface decoration with a rabies viral glycoprotein peptide was able to selectively deliver exosomal miR-29 to prevent muscle wastage by inhibiting the transcription factor Yin Yang 1 (YY1)/TGF-β3 axis [49]. Alternatively, human adipose-derived mesenchymal stem cells (ADSCs) overexpressing miR-29a inhibited TGF-β2/Smad3 signaling and reduced excessive scarring in mice after thermal injury to the skin [50]. HUMSCs-derived exosomes loaded with a miR-29b mimic suppressed expression of interleukin 1β (IL-1β)/-6, tumor necrosis factor alpha (TNF-α), and inducible nitric oxide synthase (iNOS) and reduced myocardial fibrosis when delivered through a microneedle patch to a myocardial infarction [51]. Taken together, these findings strongly suggest that members of the miR-29 family merit further consideration for therapeutic development in AF.

#### miR-146

Another candidate is miR-146 due to its role as a regulator of fibrotic TGF signals in disease progression. A study on *Schistosoma japonicum* infection-induced fibrosis concluded that the expression of exosomal miR-146a-5p in mouse and human serum negatively correlated with the severity of liver fibrosis [52]. Post-myocardial infarction, exosomal miR-146a from transfected ADSCs directly suppressed early growth response factor 1 (EGR1) activation and IL-1β/-6 and TNF-α expression due to inhibition of the toll-like receptor 4 (TLR4)/NF-κB pathway [53]. Similarly, exosomes secreted by cardiac-resident mesenchymal progenitor cells (CPCs) with miR-146a-5p prevented drug-induced cardiotoxicity, left ventricular scarring, and cardiac dysfunction [54]. In a rat urethral fibrosis model induced by TGF-β1, miR-146a-enriched exosomes derived from TNF-α-treated HUMSC suppressed urethral stricture by reduction of IL-1β/-6, IL-1 receptor-associated kinase 1 (IRAK1), TNF receptor-associated factor 6 (TRAF6), and NF-κB levels in target cells [55]. Thus, the broad anti-fibrotic effects of miR-146a in liver, heart, and urethral fibrosis, make it a promising candidate for AF therapy.

#### miR-148/-152

The members of the miR-148/miR-152 family share the same stem-loop structure prior to scission and target pathways responsible for cell inflammation, proliferation, differentiation, and survival [56, 57]. Exosomes from BMSCs with miR-148a-5p mitigated liver fibrosis in a mouse model by downregulating Smad4 in stellate cells, as confirmed by reduced TGF-β1 and tissue inhibitor of metalloproteinase 1 (TIMP-1) [58]. BMSCs-derived exosomes carrying miR-148a reduced liver scarring by affecting Kruppel-like factor 6 (KLF6) to suppress pro-inflammatory macrophages and promote anti-inflammatory macrophages *via* inhibition of the signal transducer and activator of transcription 3 (STAT3) axis [59]. In patients with acute myocardial infarction, the downregulated exosomal miR-152-5p from serum correlates strongly as a biomarker for cardiac fibrosis [60]. Inhibiting inflammatory pathways using miR-148/152 offers another novel alternative to attenuate the overproduction of ECM components during scar development.

#### miR-214

Another critical regulator of fibrosis is miR-214. It is a recognized modulator of inflammation-mediated excessive accumulation of ECM proteins due to overexpression of CTGF [61]. CTGF expression in activated hepatocytes from a liver fibrosis model was inhibited by hepatic stellate cell (HSC)-derived exosomes with enhanced levels of miR-214 [62]. Endometrial lesions were cured by transfected ectopic endometrial stromal cells (ESCs) to enrich miR-214 levels, resulting in decreased expressions of fibrosis-associated proteins including collagen and CTGF [63]. Notably, another study recorded lower circulating levels of miR-214 serving as a pathologic marker in endometriosis [64]. These findings indicate that the anti-fibrotic effects of miR-214 are mediated by the down-regulation of CTGF-activated pathways that play a fibrogenic role in multiple organ systems.

#### miR-223

miR-223 exerts anti-inflammatory and anti-fibrotic effects on ECM-producing genes as a part of resident cell activation during failed tissue repair [65]. HUMSCs-derived exosomes alleviated silica-induced lung fibrosis *via* local enhancement of miR-223-3p to repress macrophage-directed nucleotide-binding domain, leucine-rich repeat, and pyrin domain-containing protein 3 (NLRP3)-related inflammation [66]. Exosomes from miR-223 mimic transfected HUMSCs protected against cardiomyocyte inflammation and apoptosis *via* the TGF/P53/S100A9 axis to alleviate scarring post-infarction [67]. The correlation with inflammation was confirmed by the transfer of biochemically primed macrophage exosomes to hepatocytes to inhibit miR-223 resulting in activation of transcriptional activator with PDZ-binding motif (TAZ), NLRP3, and C-X-C motif chemokine ligand 10 (CXCL10) [68]. 

### Pro-fibrotic miR-21

**Fig. 3:**
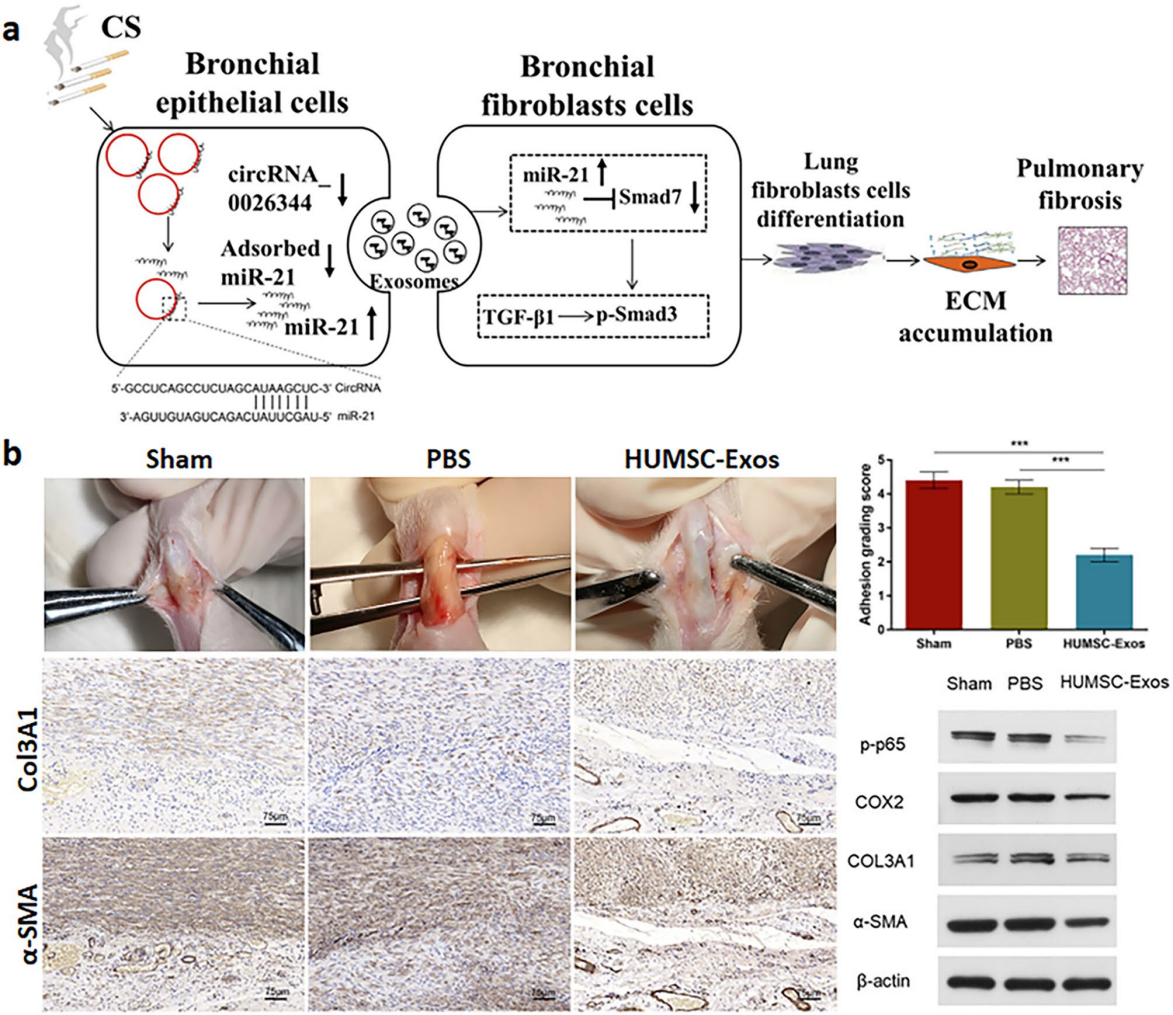
Pro-fibrotic effects of exosomal micro ribonucleic acid 21 (miR-21) in fibrous tissues. (**A**) A schematic diagram showing that circRNA-0026344 induces aberrant epithelium-fibroblast cross-talk in airway epithelial cells exposed to cigarette smoke (CS) by regulating miR-21 and causing pulmonary fibrosis. circRNA: circular ribonucleic acid, Smad: suppressor of mothers against decapentaplegic homolog, TGF-β1: transforming growth factor beta 1, ECM: extracellular matrix [71]. Copyright 2021, Elsevier B.V. (**B**) Inhibition of tendon adhesion by delivering low-abundance miR-21a-3p *via* human umbilical cord mesenchymal stem cell-derived exosomes (HUMSC-Exos). PBS: phosphate buffered saline, Col3A1: collagen type 3A1, α-SMA: alpha smooth muscle actin, COX2: cyclooxygenase-2 [78]. Copyright 2020, Yao et al

miR-21 plays an important role in fibrosis by modulating multiple fibrotic genes involved in activating resident cell and immunomodulated ECM secretion [69]. Arsenic exposure in human bronchial epithelial (HBE) cells caused secretion of miR-21-rich exosomes that in fibroblasts activated the phosphatase and tensin homolog (PTEN)/protein kinase B (Akt) signal to promote glycolytic-myofibroblast differentiation [70]. Pulmonary fibrosis (PF) from cigarette smoke is partly due to subsequent enrichment of HBE exosomes with miR-21, which, when taken up by fibroblasts, modulates the TGF-β1/Smad3 pathway to increase collagen and α-SMA expression (Fig. 3A) [71]. Further, serum exosomal miR-21-5p was reported as a strong indicator of IPF [72]. The fibrotic character of miR-21-5p was recorded as both a biomarker in pneumonia patients’ BALF fibrocytes and a therapeutic agent in controlling TGF-β1 downstream genes [73]. 

Senescence from a high-glucose diet maintained higher levels of miR-21 in tubular cells-derived exosomes that facilitated EMT and the peroxisome proliferator-activated receptor alpha (PPARα)/hypoxia-inducible factor 1 alpha (HIF-1α) pathway in kidney fibrosis [74]. In UUO-induced renal fibrosis, exosomes from TGF-β1-treated neighboring rat kidney tubular epithelial (NRK)-52E cells stimulated miR-21/PTEN/Akt in fibroblasts, leading to scar formation [75]. Notably, abolishing miR-21-5p packaging in mesenchymal stem cells (MSCs)-derived exosomes attenuated its metabolic reprogramming toward aerobic glycolysis *via* the inhibition of phosphofructokinase muscle isoform (PFKM), a rate-limiting enzyme of glycolysis in tubular epithelial cells (TECs) [76]. Besides organ fibrosis, the pro-fibrotic effects of miR-21 could be observed in tendon and epidural fibrosis [77]. For example, exosomes secreted by HUMSCs with low miR-21a-3p avoided p65 activation and subsequent myofibroblast differentiation causing tendon adhesion (Fig. 3B) [78]. Thus, miR-21 plays an essential regulator of fibrosis across various tissues, and inhibiting its activity has the potential to lessen the severity of fibrotic conditions.

### Other potential MiRNAs

Table 3 is an additional list of exosomal miRNAs demonstrating both anti- and pro-fibrotic effects. It is increasingly apparent that exosomal miRNAs secreted by MSCs either naturally or under metabolic priming, pack pharmaceutically beneficial miRNAs. For example, miR-192-5p [79], miR-150-5p [80], and miR-181d [81] were highly expressed in therapeutically relevant stem cells-derived exosomes. Injection of these exosomes reduced fibrotic scars in vital organs, presumably by dampening inflammatory signals on resident cells. It should be noted that the desired anti-fibrotic effects were also achieved by bioengineering exosomal miR-122 [82, 83] and miR-181-5p [84]. Qu and colleagues claim that approximately 3.5 times more engineered exosomal miR-181 was transported into recipient HSCs than with naturally occurring exosomes. These exosomes prevented myofibroblast activity *via* STAT3/B-cell lymphoma 2 (Bcl-2) [84]. Interestingly, hypoxic cues mimicking the molecular dysfunction observed during infarction delivered to induced pluripotent cardiomyocytes increased the levels of exosomal miR-181a, which suggests that a therapy involving antagomirs to miR-181a might alleviate cardiac hypertrophy [85]. Similarly, hypoxic stress in renal fibrosis triggered epithelial cells to pack more miR-150 in exosomes that eventually were taken up by mouse fibroblasts to sustain soft tissue scarring [86, 87]. Exosomal miR-122 and miR-192 from non-alcoholic fatty liver disease (NAFLD) patient blood mimicked the palmitic acid treatment response on the Huh-7 cell exosomes, reportedly increasing COL1A1, α-SMA, and TGF-β1 levels in recipient fibroblasts [88, 89]. Citreoviridin, a mycotoxin that triggers liver damage, influences the exposed hepatocytes to encapsulate extra miR-181a-2-3p to activate HSCs by mitochondrial calcium overloading [90]. Unlike the candidate miRNAs in Tables 1 and 2, these miRNAs do not produce outcomes that can be categorized as either pro-fibrosis or anti-fibrosis. Therefore, these are not viewed as priority candidates for anti-AF therapy. The limited variety in primary miRNA literature and variation in parent and recipient cells studied mandate additional research prior to any inference on its global role in soft tissue fibrosis.

### Risk of bias assessment

**Fig. 4:**
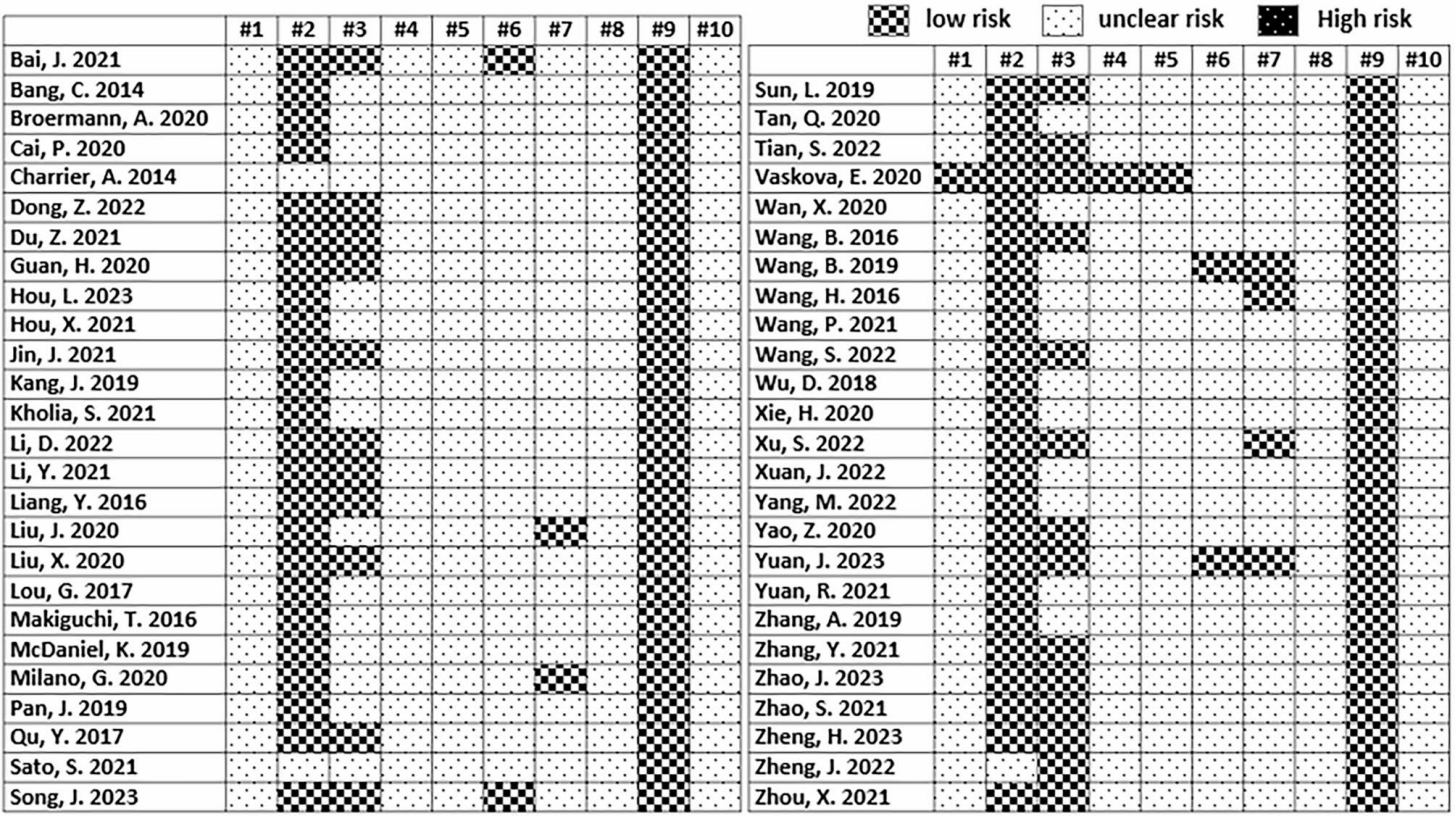
Systemic Review Centre for Laboratory Animal Experimentation (SYRCLE’s) risk of bias tool for animal studies: (1) selection bias/sequence generation, (2) selection bias/baseline characteristics, (3) selection bias/allocation concealment, (4) performance bias/random housing, (5) performance bias/blinding, (6) detection bias/random outcome assessment, (7) detection bias/blinding, (8) attrition bias/incomplete outcome data, (9) reporting bias/selective outcome reporting, and (10) other sources of bias

The risk of bias for animal intervention studies was assessed using a Systemic Review Centre for Laboratory Animal Experimentation (SYRCLE’s) tool comprising 10 checklist items: (1) selection bias/sequence generation, (2) selection bias/baseline characteristics, (3) selection bias/allocation concealment, (4) performance bias/random housing, (5) performance bias/blinding, (6) detection bias/random outcome assessment, (7) detection bias/blinding, (8) attrition bias/incomplete outcome data, (9) reporting bias/selective outcome reporting, and (10) other sources of bias [91]. A total of 51 references were processed and summarized in Fig. 4. Overall, most of the references were verified by low risk of bias in baseline characteristics (96.1%), allocation concealment (49.0%), and selective outcome reporting (100%) or unclear risk.

## Conclusion

**Fig. 5:**
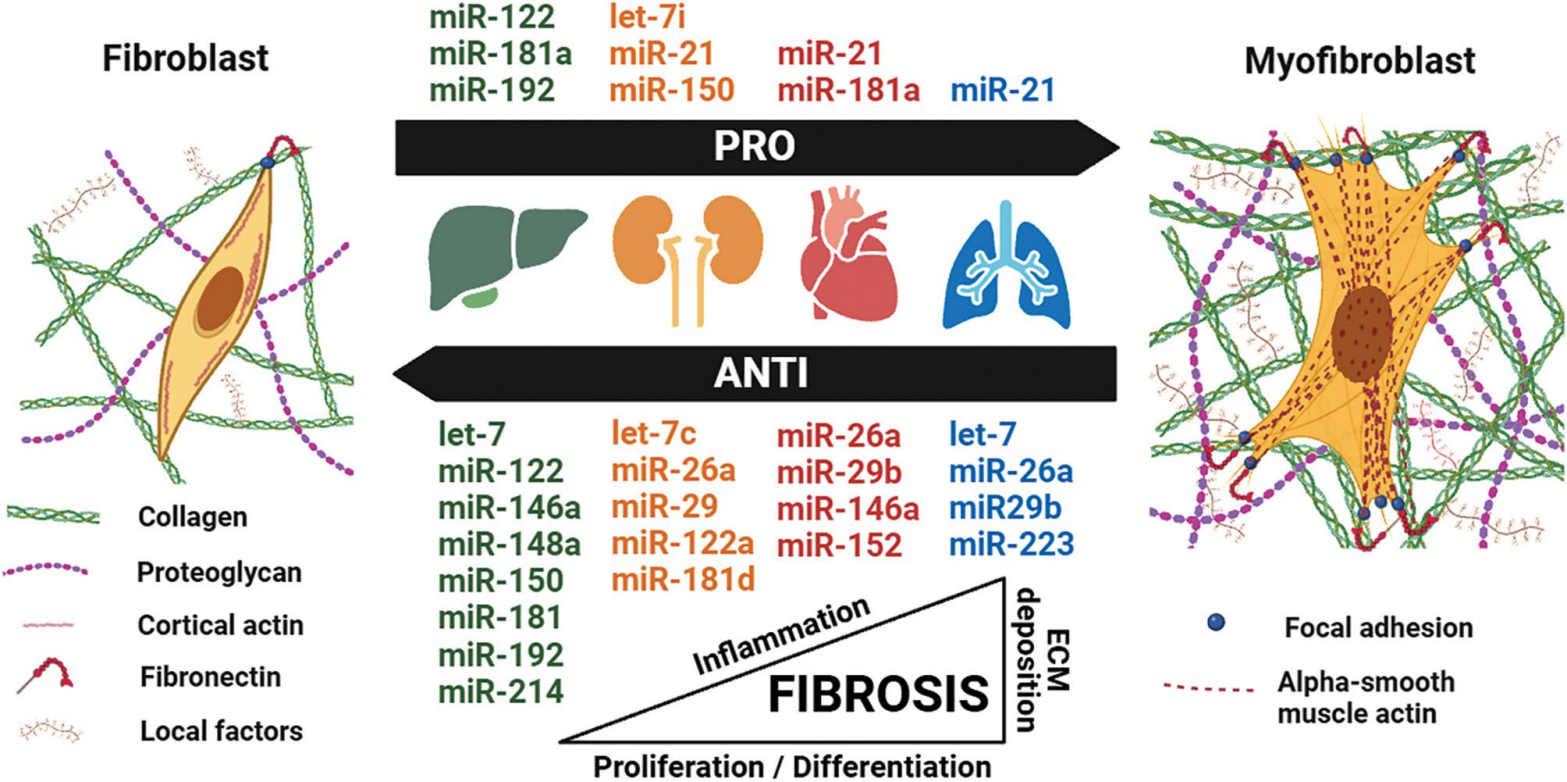
Summarized candidate micro ribonucleic acids (miRs/miRNAs) for arthrofibrosis treatment. PRO: pro-fibrosis, ANTI: anti-fibrosis, ECM: extracellular matrix

In this comprehensive review, we summarized the literature on the roles of exosomal miRNAs in fibrosis to identify candidates for intraarticular AF therapy (Fig. 5). The common mechanisms such as excessive ECM deposition, myofibroblast activation, exaggerated inflammatory response, traumatic tissue injury, and tissue stiffness underlying fibrogenesis in multiple organ systems and articular joints can allow for predicting the translatability of established antifibrotic miRNAs in AF. From this survey, we can conclude that the most eligible candidates include members of the let-7, miR-26, miR-29, miR-146, miR-148/-152, miR-214, and miR-223 families that oppose fibrogenic pathways driven by dysregulated TGF/CTGF expression. These sequences are generally enriched in exosomes derived from pluripotent cells (e.g., BMSCs), which could serve as a source of exosomes for AF therapy. Another option is to introduce single or multiple synthetic miRNAs into joint cells using manufactured exosomes (e.g., lipid nanoparticles) as carriers [92]. Delivery can be targeted to myofibroblasts or immune cells by incorporating ligands or antibodies to cell surface receptors into lipid membranes [93]. A similar approach could be used to deliver synthetic antagomirs to pro-fibrotic miRs (e.g., miR-21).

Thus, there appear to be a number of miR-based strategies that can be pursued to improve AF outcomes. In vitro and in vivo studies are needed to sort out which of these options to pursue for advanced development and clinical testing. These should include evaluation of excipients (e.g., hydrogels) to facilitate intraarticular retention of exosomes, thereby minimizing the need for multiple joint injections that tend to promote synovial inflammation and increase risks for infection.

## Data Availability

The datasets generated during and/or analyzed during the current study are available from the corresponding author on reasonable request.
